# The cost-effectiveness of medical versus surgical androgen deprivation therapy for advanced prostate cancer in Tanzania: a Markov model-based analysis

**DOI:** 10.3389/fpubh.2026.1907445

**Published:** 2026-09-18

**Authors:** Obadia Nyongole, Malale Tungu, Alphoncina Kagaigai, Amani Anaeli, Amisa Tindamanyile, Prosper Njau, Mucho Mizinduko, David Urassa, Bruno Sunguya, Gasto Frumence

**Affiliations:** 1 Department of Surgery, Muhimbili University of Health and Allied Sciences, Dar es Salaam, Tanzania; 2 Department of Development Studies, Muhimbili University of Health and Allied Sciences, Dar es Salaam, Tanzania; 3 School of Pharmacy, Muhimbili University of Health and Allied Sciences, Dar es Salaam, Tanzania; 4 Ministry of Health, Dodoma, Tanzania; 5 Department of Epidemiology and Biostatistics, Muhimbili University of Health and Allied Sciences, Dar es Salaam, Tanzania; 6 Department of Community Health, Muhimbili University of Health and Allied Sciences, Dar es Salaam, Tanzania

**Keywords:** cost-effectiveness, medical ADT, surgical ADT, advanced prostate cancer, quality-adjusted life years (QALYs)

## Abstract

**Background:**

Androgen deprivation therapy (ADT) is a recommended treatment for patients with advanced prostate cancer, mostly for palliative purposes. In limited resource settings, decision-making about palliative treatment should be guided by the overall survival benefit of one option over another, patient life expectancy, quality of life, and cost.

**Purpose:**

This study aimed to evaluate the cost-effectiveness of medical androgen deprivation therapy compared to surgical androgen deprivation therapy among patients with advanced prostate cancer in Tanzania.

**Methods:**

A model-based economic evaluation study design, was conducted using retrospective patient records from tertiary hospitals in Tanzania and published literature. A Markov model with a five-year time horizon was developed to simulate disease progression among patients receiving either medical ADT or surgical ADT. Health states included local recurrence, asymptomatic metastatic disease, symptomatic metastatic disease, death from prostate cancer, and death from other causes. Costs were estimated from provider and societal perspectives and expressed in US$. Health outcomes were measured in quality-adjusted life years (QALYs). Incremental cost-effectiveness ratios (ICERs) were calculated and compared against a willingness-to-pay threshold based on Tanzania’s gross domestic product (GDP) per capita (US$1,099) as the cost-effectiveness threshold. Alternative willingness-to-pay thresholds, including three times GDP per capita (US$3,580), were explored in sensitivity analyses. Probabilistic sensitivity analysis using Monte Carlo simulation was performed to assess uncertainty.

**Results:**

Medical ADT generated higher costs and greater health benefits than surgical ADT. The total cost of medical ADT was estimated at US$50,639.98 compared with US$6,917.51 for surgical ADT. Medical ADT produced a cumulative cohort-level effectiveness equivalent to 105.63 QALYs compared with 4.06 QALYs for surgical ADT, resulting in an incremental gain of 101.58 QALYs. The ICER for medical ADT was US$430.44 per QALY gained, which remained substantially below the selected willingness-to-pay threshold of US$1,099 and alternative threshold of US$3,580 per QALY. Probabilistic sensitivity analyses demonstrated that medical ADT remained cost-effective across a wide range of parameter assumptions.

**Conclusion:**

Medical ADT was more costly but also more effective than surgical ADT for managing advanced prostate cancer in Tanzania. The estimated ICER remained below the willingness-to-pay threshold, indicating that medical ADT represents a cost-effective treatment strategy. Further studies incorporating local utility estimates and long-term survival outcomes are warranted to strengthen economic evidence for prostate cancer management in Tanzania.

## Introduction

Prostate cancer is the second most commonly diagnosed cancer among men globally and a leading cause of cancer-related mortality. According to GLOBOCAN 2020 estimates, approximately 1.4 million new prostate cancer cases and over 375,000 deaths occurred worldwide, with a rapidly increasing burden in low- and middle-income countries (LMICs) due to population ageing and improved detection (1). In sub-Saharan Africa (SSA), prostate cancer frequently presents at advanced stages because of delayed diagnosis, limited screening opportunities, and restricted access to specialized oncology services (2). In Tanzania, studies have reported that the majority of patients present with locally advanced or metastatic disease, substantially limiting opportunities for curative treatment and increasing healthcare costs (3).

Androgen deprivation therapy (ADT) remains the cornerstone of treatment for advanced prostate cancer. ADT can be achieved through either surgical castration (orchiectomy) or medical suppression using luteinizing hormone-releasing hormone agonists and anti-androgen medications. Although both approaches effectively suppress testosterone and delay disease progression, they differ considerably in terms of cost, reversibility, treatment adherence, adverse effects, and patient acceptability (4–6).

Patients with advanced prostate cancer (APC) in developing countries have limited treatment options other than androgen deprivation therapy (ADT) which can be medical, surgical, or both (7, 8). Few patients have access to radiotherapy, mainly external beam radiation and surgery (salvage prostatectomy), even though centers for it are limited compared to the magnitude of cancer in Sub-Saharan Africa (SSA) (1, 2, 7, 9). In low- and middle-income countries. It has been documented that patients seek healthcare when they have advanced cancer, thereby limiting treatment options to ADT (2, 7, 10, 11). Long-term use of ADT is costly because of the cost of therapy and the cost of treating adverse events (AEs) (12).

Quality-adjusted life years (QALYs) are important for those patients who are on ADT due to APC (12–14). Although metastatic prostate cancer is incurable, medical and surgical androgen suppression can palliate its symptoms and may prolong survival (4, 12). There is little basis for determining which of the available androgen suppression treatments represents the best value (4, 13). In resource-constrained health systems like Tanzania, clinical effectiveness alone is insufficient to guide treatment decisions. Economic evaluation provides an important framework for assessing whether additional health benefits generated by an intervention justify the additional costs incurred. Cost-effectiveness analysis is particularly relevant in LMICs, where healthcare budgets are limited and competing health priorities require efficient allocation of resources (15). Several studies conducted in high-income countries have demonstrated that medical ADT may be cost-effective despite its higher acquisition costs because of gains in QALYs and improvements in patient preferences and quality of life (4, 14, 16, 17). However, evidence from SSA remains scarce, and findings from high-income settings may not be directly transferable to Tanzania due to differences in healthcare financing, treatment costs, health system capacity, and patient characteristics.

In Tanzania, limited evidence is available regarding the costs of the management of prostate cancer (2, 3). To address the existing knowledge gap, this study evaluated the cost-effectiveness of medical ADT compared with surgical ADT among patients with advanced prostate cancer in Tanzania using a Markov decision-analytic model. The comparison is based on QALYs and cost-effectiveness over a 5- year timeframe. The findings are expected to inform clinical practice, health financing decisions, and prostate cancer treatment policies in Tanzania and similar resource-limited settings.

## Methods

A model-based economic evaluation using retrospective patient records and published literature of patients who were treated for prostate cancer at tertiary hospitals in the year 2022 to compare the cost-effectiveness of medical and surgical ADT among patients with advanced prostate cancer in Tanzania. Patient records with incomplete information on treatment received, cost data, or clinical outcomes required for model development were excluded from the analysis. Consequently, a complete-case analysis was undertaken. The original record-screening log did not retain a reliable count of all records excluded specifically because of missing information; consequently, the proportion of records excluded for missing data could not be retrospectively quantified. This study evaluated the cost-effectiveness of the two interventions (medical ADT and surgical ADT, comparing QALYs and the costs of treating patients with advanced prostate cancer when treated by ADT using a Markov model with two treatment arms: Medical ADT and Surgical ADT. We estimated the health effects associated with medical and surgical androgen suppression strategies for local recurrent and asymptomatic disease states. Furthermore, we used both medical and surgical ADT for symptomatic distant metastatic disease states (13, 14). TreeAge Pro software was used in the analysis of cost-effectiveness data (18).

Transition probabilities governing movement between Markov health states were derived from published natural history studies and randomized clinical trials of advanced prostate cancer, following the methodology described by Bayoumi et al. Disease progression estimates were informed by studies of recurrence after definitive therapy, local recurrence, metastatic progression, and androgen deprivation therapy. Where annual transition rates were reported, they were converted to cycle-specific transition probabilities using standard exponential conversion methods. A one-year cycle length was selected to align with the availability of annual follow-up data from Tanzanian clinical records and published literature. Annual transition probabilities were therefore applied directly without further cycle conversion.

Markov modeling was used to simulate disease progression and treatment outcomes among patients with advanced prostate cancer receiving either medical ADT or surgical ADT over a five-year time horizon. The model included the following health states: local recurrent disease, asymptomatic metastatic disease, symptomatic metastatic disease, death from prostate cancer, and death from other causes. Patients transitioned between health states annually based on probabilities obtained from published literature and patient records. A yearly cycle length was applied, and future costs and health outcomes accruing during each annual Markov cycle were discounted at an annual rate of 3%. Discounting was applied consistently to all direct medical cost categories, including treatment costs, follow-up costs, hospitalization costs where applicable, and end-of-life care, as well as to all quality-adjusted life-years accumulated throughout the model.

The five-state Markov model was selected to represent the principal stages of advanced prostate cancer progression relevant to evaluating the comparative cost-effectiveness of ADT. Although more complex models may incorporate tumour grade, treatment-specific adverse events, or additional disease substates, a parsimonious model was considered appropriate because the primary objective was to compare the overall long-term costs and health outcomes associated with surgical and medical ADT rather than to evaluate disease prognosis within specific clinical subgroups. Patients who received both medical and surgical ADT during the study period were not included in the comparative cost-effectiveness analysis because the objective was to compare mutually exclusive treatment strategies. Consequently, the Markov model was parameterized using patients who received either medical ADT alone or surgical ADT alone.

This study combined societal and provider perspectives on economic costs and epidemiological outcomes (QALYs) from the two interventions to estimate the cost per QALY gained. Utility weights used to estimate QALYs were obtained from previously published studies on advanced prostate cancer and androgen deprivation therapy. QALYs were estimated by multiplying the duration spent in each Markov health state by its corresponding health-state utility value during each annual cycle. Due to limited local utility data from Tanzania, published international utility estimates were adopted. QALYs were accumulated across all health states over the five-year time horizon, discounted at an annual rate of 3% on life years gained, and summed to obtain the cumulative expected QALYs for each treatment strategy, which were calculated based on the standard methods using WHO life tables (4, 19). QALYs reported in the analysis represent cumulative cohort-level outcomes rather than individual patient-level QALYs.

The Incremental cost-effectiveness ratio (ICER) was calculated by taking the difference in the costs of medical ADT and surgical ADT divided by their differences in the QALYs gained. The cost-effectiveness of the interventions was assessed by comparing the ICER with a willingness-to-pay (WTP) threshold based on Tanzania’s gross domestic product (GDP) per capita for 2022. The base-case analysis adopted Tanzania’s GDP per capita (US$1,099/QALY) as the cost-effectiveness threshold (20), and GDP per capita (US$3,580), were explored in sensitivity analyses. An ICER equal to or lower than this threshold meant that the intervention was cost-effective, and one higher than the threshold was regarded as not cost-effective. In line with recommendations for assessing decision uncertainty, alternative willingness-to-pay thresholds were explored in sensitivity analyses.

### Cost estimation

Costs were estimated from both the provider and societal perspectives and expressed in 2022 US$ to facilitate comparison of costs and outcomes. Cost data were obtained from hospital records, patient records, and relevant literature sources. Where necessary, costs collected in Tanzanian Shillings (TZS) were converted to US$ using the average annual exchange rate of 1 US$ = 2,327.99 TZS reported by the Bank of Tanzania for the year 2022 (21). All costs were standardized to 2022 US$ to ensure comparability across cost categories. To estimate the annual average direct cost of the treatment, a descriptive statistics analysis was carried out. This included the sum of direct medical costs such as consultation fees, investigations/laboratory tests fees, treatment, medicines, and other supplies, hospitalization costs, and direct non-medical expenses such as transport for the patient and accompanying relatives. For indirect costs, a monetary value was assigned to the number of days of work missed by cancer patients and their caregivers during the treatments. The human capital approach was used to estimate indirect costs by multiplying a patient’s daily minimum wage rate by the number of days absent from work while seeking treatment. The cost values presented in Table 1 represent annual treatment costs (direct and indirect costs) and other model input parameters used in the Markov model. The total costs reported in the results section represent cumulative discounted costs accrued over the five-year analytic horizon after accounting for disease progression, survival, and transitions between health states.

**Table 1:** Model input parameters used in the economic evaluation.

Model input parameter	Base-case value	Range/uncertainty	Distribution	Source	Basis/assumption
Model structure					
Cycle length	1 year	Fixed	Fixed	Present study	Consistent with annual follow-up data
Time horizon	5 years	Lifetime scenario	Fixed	Present study	Study objective
Discount rate for costs and QALYs	3% annually	0–5%	Fixed	Bayoumi et al.; CHEERS recommendations	Standard economic evaluation practice
Initial cohort—medical ADT	1,024 patients	Fixed	Fixed	Present study	Observed medical ADT-only cohort
Initial cohort—surgical ADT	352 patients	Fixed	Fixed	Present study	Observed surgical ADT-only cohort
Transition probabilities					
Local recurrence → asymptomatic metastatic disease	0.1136 per year	±20%	Beta^*^	Pound et al. (31); Bayoumi et al.	Annual probability derived from reported monthly probability of 1.0%
Asymptomatic metastatic disease → symptomatic metastatic disease	0.6982 per year	±20%	Beta^*^	Cowen et al. (32); Bayoumi et al.	Annual probability derived from reported monthly probability of 9.5%
Symptomatic metastatic disease → death from prostate cancer	0.4099 per year	±20%	Beta^*^	Crawford et al. (33); Byar (34, 35); Bayoumi et al.	Annual probability derived from reported monthly probability of 4.3%
Death from other causes	Age-specific mortality	95% CI from life tables	Beta^*^	WHO Life Tables	Competing mortality
Health-state utility values					
Local recurrence/progression-free disease	0.830	0.66–1.00	Beta	Barbier et al. (14)	EQ-5D-based utility for progression-free metastatic hormone-sensitive prostate cancer
Asymptomatic metastatic disease	0.800	0.64–0.96	Beta	Wang et al. (12)	EQ-5D-5L utility for metastatic castration-sensitive prostate cancer
Symptomatic metastatic disease	0.716	0.57–0.86	Beta	Wang et al. (12)	Utility for metastatic castration-resistant prostate cancer
Terminal illness	0.400	0.32–0.48	Beta	Barbier et al. (14)	Utility for terminal progressive disease
Death	0.000	Fixed	Fixed	Standard economic evaluation	Absorbing state
Treatment costs					
Annual medical ADT	US$2,940.00 per patient-year	±20%	Gamma^*^	Tanzanian hospital/patient records	Local empirical costing
Surgical ADT	US$1,205.20 per patient	±20%	Gamma^*^	Tanzanian hospital/patient records	Local empirical costing
Health-state/progression costs	Not numerically recoverable from archived model	–	–	Original costing/model records	Included in original model but numerical state-specific inputs were not available for independent retrieval
Hospitalization costs	Included where documented	–	–	Hospital records	Incorporated into disease-management costs where documented
Follow-up costs	Included	–	–	Hospital/patient records	Costs associated with disease progression and follow-up
Laboratory investigations	Included	–	–	Hospital/patient records	Costs associated with disease monitoring
Imaging	Included	–	–	Hospital/patient records	Costs associated with disease monitoring
Supportive care	Included	–	–	Hospital/patient records	Costs associated with disease progression
End-of-life care	Included	–	–	Hospital/patient records	Costs associated with terminal management
Infrastructure/capital costs	Excluded	–	Fixed	Present study	Reliable patient-level allocation was not feasible
Currency and valuation					
Exchange rate	TZS 2,327.99 = US$1	Fixed	Fixed	Bank of Tanzania	Average annual 2022 exchange rate
Cost year	2022 US$	Fixed	Fixed	Present study	All costs standardized to 2022 US dollars

ADT, androgen deprivation therapy; PSA, probabilistic sensitivity analysis. For Beta distributions, parameter 1 represents α and parameter 2 represents β. For Gamma distributions, parameter 1 represents the shape and parameter 2 represents the scale. The PSA used 100,000 Monte Carlo simulations. Where empirical standard errors or confidence intervals were unavailable, uncertainty was represented using a ± 20% range around the base-case estimate. The parameters presented for such inputs are reconstructed from the reported base-case values and uncertainty assumptions and should therefore not be interpreted as directly retrieved from the original TreeAge Pro model. ^*^Retain the terminal-illness utility only if this utility was actually included as a model state in the original TreeAge implementation. Where original model inputs could not be independently retrieved following expiry of the TreeAge Pro license, the manuscript reports the available numerical inputs and their sources and explicitly identifies the remaining parameters as limitations of model reproducibility.

Hospitalization costs were included in the costing analysis where they were documented in patient records and were incorporated into disease-management costs during the Markov simulation. Infrastructure and capital costs, including building depreciation, utilities, and administrative overheads, were excluded because reliable allocation of these shared costs to individual prostate cancer patients was not feasible using available hospital financial records. The costing analysis therefore focused on direct medical costs that varied according to treatment received.

The annual costs presented in Table 1 represent the direct costs associated with ADT itself. During the Markov simulation, patients accumulated additional costs associated with disease progression, including follow-up visits, laboratory investigations, imaging, supportive care, hospitalization, and end-of-life management. Consequently, the cumulative five-year costs reported in the Results represent the total expected costs accrued across all health states rather than simply five times the annual treatment cost.

The model input parameters included annual treatment costs, transition probabilities between health states, utility values, mortality probabilities, and discount rates. Transition probabilities were obtained from published studies and used to simulate movement between disease states during each annual cycle. Utility values were assigned to each health state to reflect differences in health-related quality of life and were used to estimate QALYs. Cost and utility parameters were varied in sensitivity analyses to evaluate uncertainty in model outcomes.

Transition probabilities were derived from published natural history studies and randomized clinical trials identified by Bayoumi et al. (4). Because the original studies reported disease progression as transition rates or survival times rather than explicit annual transition probabilities, transition probabilities were derived following the approach described by Bayoumi et al. Health-state utility values were obtained from recent EQ-5D-based economic evaluations by Wang et al. and Barbier et al. (12, 14). Where measures of parameter uncertainty were unavailable, a ± 20% variation around the base-case value was assumed for deterministic sensitivity analyses.

### Justification for model input parameters

Tanzania currently lacks published longitudinal data describing disease progression among patients receiving androgen deprivation therapy, as well as preference-based health utility estimates for advanced prostate cancer. Consequently, transition probabilities and health-state utility values were obtained from internationally published studies that employed comparable disease pathways, treatment strategies, and validated health-related quality-of-life instruments. Preference was given to studies evaluating patients with advanced or metastatic prostate cancer receiving ADT, as these populations most closely resembled the Tanzanian study population. Although these parameters were derived from high-income settings, the underlying biological progression of advanced prostate cancer is expected to be similar across populations, while differences related to healthcare delivery and supportive care were explored through sensitivity analyses.

### Sensitivity analysis

Probabilistic sensitivity analysis (PSA) was conducted in the original TreeAge Pro model using 100,000 Monte Carlo simulations to assess uncertainty in model parameters. Beta distributions were assigned to transition probabilities and health-state utility values, while gamma distributions were assigned to cost parameters. Where empirical measures of uncertainty were unavailable, plausible parameter ranges were used to represent parameter uncertainty. Because the original TreeAge Pro model file and detailed distribution-parameter outputs were no longer accessible at the time of manuscript revision, the exact alpha, beta, shape and scale parameters used in the original simulations could not be independently retrieved. This limitation is acknowledged in the interpretation of the PSA findings. During each iteration, parameter values were randomly sampled from their respective distributions and the ICER recalculated. Results were summarized using cost-effectiveness acceptability curves and cost-effectiveness scatter plots. We calculated the average annual cost per case and QALY gained, and for comparison purposes, cost per person protected and cost per death averted as a point estimate (the mean of 100,000 iterations) with uncertainty represented by the cut-off value where the 5 and 95% of simulation outputs fall (90% credible interval). Sensitivity analysis was conducted to rank and quantify inputs (e.g., cost or useful life) according to the amount of swing (positive or negative) they caused on the outputs, i.e., cost-effectiveness indicators, cost per QALY gained (12, 22).

Treatment outcomes (local recurrence of prostate cancer, asymptomatic distant metastatic prostate cancer, symptomatic distant metastatic prostate cancer, death from prostate cancer, and death from other causes) to give QALYs across medical and surgical ADT options (4, 13, 22).

Patients with advanced prostate cancer entered the model and received either medical ADT or surgical ADT. During each annual cycle, patients could remain in their current health state, progress to a more advanced disease state, die from prostate cancer, or die from other causes. Costs and utility values were assigned to each health state, and cumulative costs and QALYs were estimated over a five-year time horizon as indicated in Figure 1.

**Figure 1:**
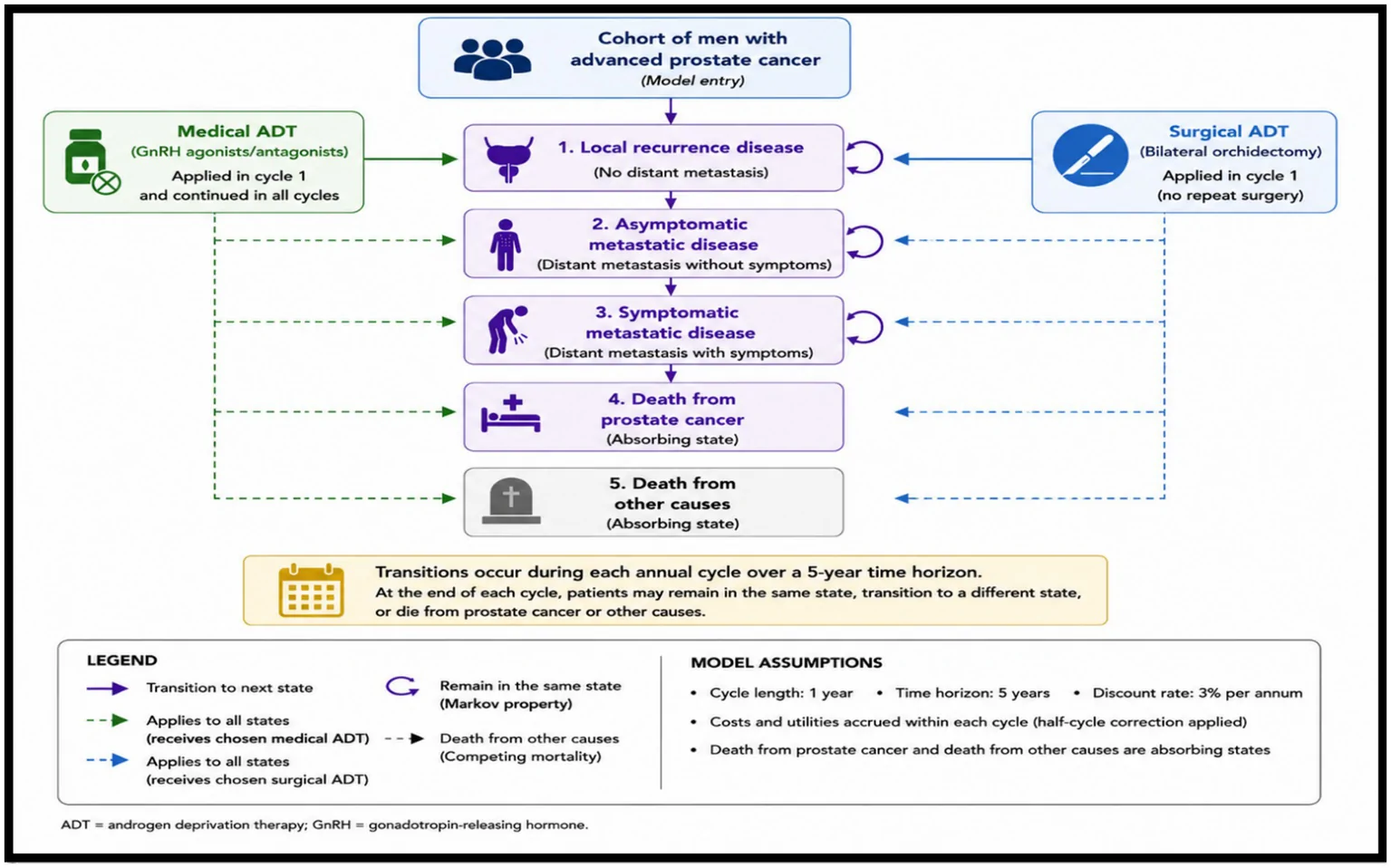
Five-state transition Markov model for patients with advanced prostate cancer receiving ADT.

### Scenario analyses

To evaluate the robustness and transferability of the model input parameters, scenario analyses were undertaken using alternative health-state utility estimates and disease progression evidence identified from published economic evaluations of advanced prostate cancer. Utility values reported by Wang et al. and Barbier et al. were compared with the base-case values adopted in the model (12, 14). Alternative disease progression evidence identified from Bayoumi et al. and the underlying natural history studies was also reviewed to assess the consistency of disease progression assumptions across published models (4). These analyses were intended to evaluate the influence of alternative parameter sources on the interpretation of the cost-effectiveness results.

To evaluate the robustness of model assumptions, alternative published utility estimates and disease progression evidence were identified from the literature. These alternative parameter sources were compared with the base-case inputs to assess whether different evidence sources were likely to materially influence the interpretation of the cost-effectiveness results. The alternative model inputs considered are summarized in Table 2.

**Table 2:** Alternative published utility values and disease progression evidence considered in scenario analyses.

Parameter	Base-case source	Alternative source	Reason for comparison	Expected impact on model
Utility: local recurrence	Barbier et al. (14)	Wang et al. (12)	Both report EQ-5D-based utilities for advanced prostate cancer	Minimal change in QALYs
Utility: symptomatic metastatic disease	Wang et al. (12)	Barbier et al. (14)	Alternative published utility estimate	Moderate reduction in QALYs
Disease progression pathway	Bayoumi et al. (4)	Pound et al. (31); Cowen et al. (32)	Alternative natural history evidence	Minimal change in disease progression assumptions
Background mortality	WHO Life Tables	WHO Life Tables	Standard mortality source	No expected impact

Deterministic sensitivity analyses were performed using the parameter ranges presented in Table 1. In addition, scenario analyses were undertaken by comparing the base-case utility values and disease progression assumptions with alternative published estimates summarized in Table 2 to evaluate the robustness of the model conclusions.

## Results

This study was preceded by a case notes (records) review of patients who were treated for prostate cancer in 2022 at tertiary hospitals in Tanzania. The mean age of patients in 2022 was 71.88 years with a standard deviation of 10.64. The majority, 1,020 (79.4%) of our patients had Pre-treatment PSA levels above 20 ng/mL. From the reviewed document, 82.5% of our patients had clinical stage cT3-cT4 of prostate cancer. Most 616 (66.8%) of our patients had high-risk/poorly differentiated prostate cancer with a Gleason score of >8. Androgen deprivation therapy (ADT) was the main treatment option, with medical androgen therapy alone dominating in 1024 (68.1%) patients. The majority of participants (1,207; 82.8%) were covered by a health insurance scheme, while 13.4% contributed through cost-sharing arrangements. Only a small proportion paid entirely out of pocket (0.9%), whereas 2.9% received fee exemptions or waivers. This high level of financial protection may have reduced direct financial barriers to accessing prostate cancer treatment, as presented in Table 3.

**Table 3:** Baseline information of patients on ADT variables.

Demographic characteristics	Description	Total (%)
Age in years	50–60	353 (23.5)
	61–70	795 (52.9)
	71–80	265 (17.6)
	>80	90 (6.0)
Education	Never went to school	89 (5.9)
	Primary education	884 (58.8)
	Secondary education	265 (17.6)
	University education	265 (17.6)
Marital status	Married	1,150 (76.5)
	Not married	265 (17.6)
	Widowed	88 (5.9)
Employment status	In service	265 (17.6)
	Retired	973 (64.7)
	Peasants	265 (17.6)
Treatment type		
ADT	Androgen deprivation therapy—Medical alone	1,024 (68.1)
	Androgen deprivation therapy—Surgical alone	352 (23.4)
	Medical and surgical ADT	127 (8.5)
Payment methods	Insurance	1,207 (82.8)
	Cost sharing	196 (13.4)
	Out of pocket	13 (0.9)
	Exemption/waiver	42 (2.9)
Clinical characteristics	Clinical stage cT1-cT2a	23 (5.6)
	Clinical stage cT2b-cT2c	49 (11.9)
	Clinical stage cT3-cT4	339 (82.5)
	Less than 6 months	230 (17)
	More than 6 months	1,123(83)
Pre-treatment PSA levels	<10 ng/mL	163 (12.7)
	10 ng/ml > PSA < 20 ng/ml	101(7.9)
	PSA > 20 ng/ml	1,020 (79.4)
Histological type	Adenocarcinoma	1,314 (99.5)
	Transitional cell carcinoma	2 (0.1)
	Lymphoma	1(0.1)
	Sarcoma	4 (0.3)
Gleason grade	Gleason score <6 (low risk/well-differentiated)	70 (7.6)
	Gleason score = 7 (Intermediate risk/Moderate differentiated)	236 (25.6)
	Gleason score >8 (High risk/Poorly differentiated)	616 (66.8)

Although 8.5% of patients received both medical and surgical ADT during routine clinical care, these patients were excluded from the economic evaluation because they did not represent a distinct treatment strategy suitable for direct comparison.

We estimated the health effects associated with medical and surgical androgen suppression strategies for local recurrent and asymptomatic disease states. Furthermore, we used both medical and surgical ADT for symptomatic distant metastatic disease states. Medical ADT is more costly as well as more effective compared to Surgical ADT. The ICER of US$430.44 per QALY gained was below the selected WTP threshold. Medical ADT generated greater quality-adjusted health benefits than surgical ADT while incurring higher treatment costs. The resulting ICER remained below the selected willingness-to-pay threshold, indicating that medical ADT is a cost-effective treatment strategy for advanced prostate cancer in Tanzania. The effectiveness values reported in Table 4 represent cumulative cohort-level QALYs generated by the model. In addition, the costs reported in Table 4 represent cumulative discounted costs accrued over the five-year time horizon, whereas the values reported in Table 1 represent annual model input costs. The results have been summarized in Table 4.

**Table 4:** An incremental cost-effectiveness (ICER) ratio of medical ADT being its value over surgical ADT.

Category	Dominance	Strategy	Cost	Incr Cost	Eff	Incr Eff	ICER	NMB
All (no dominance)	Undominated	Surgical ADT	6917.51		4.06			7603.02
All (no dominance)	Undominated	Medical ADT	50639.98	43722.48	105.63	101.58	430.44	327519.7

NMB, net monetary benefit; WTP, willingness-to-pay. NMB values were calculated using a WTP value of US$3,580 per QALY gained, corresponding to the alternative GDP-based WTP scenario examined in the analysis. The base-case WTP scenario was US$1,099 per QALY gained.

The cost-effectiveness analysis demonstrated that medical ADT generated higher costs and greater health benefits than surgical ADT. The total expected discounted cost of medical ADT was US$50,639.98 compared with US$6,917.51 for surgical ADT. Medical ADT generated greater effectiveness, resulting in an incremental cost-effectiveness ratio (ICER) of US$430.44 per QALY gained. As shown in Figure 2, medical ADT was located in the north-east quadrant of the cost-effectiveness plane, indicating that it was more costly but also more effective than surgical ADT. The estimated ICER remained below the selected WTP threshold, suggesting that medical ADT is a cost-effective treatment strategy for advanced prostate cancer in Tanzania.

**Figure 2:**
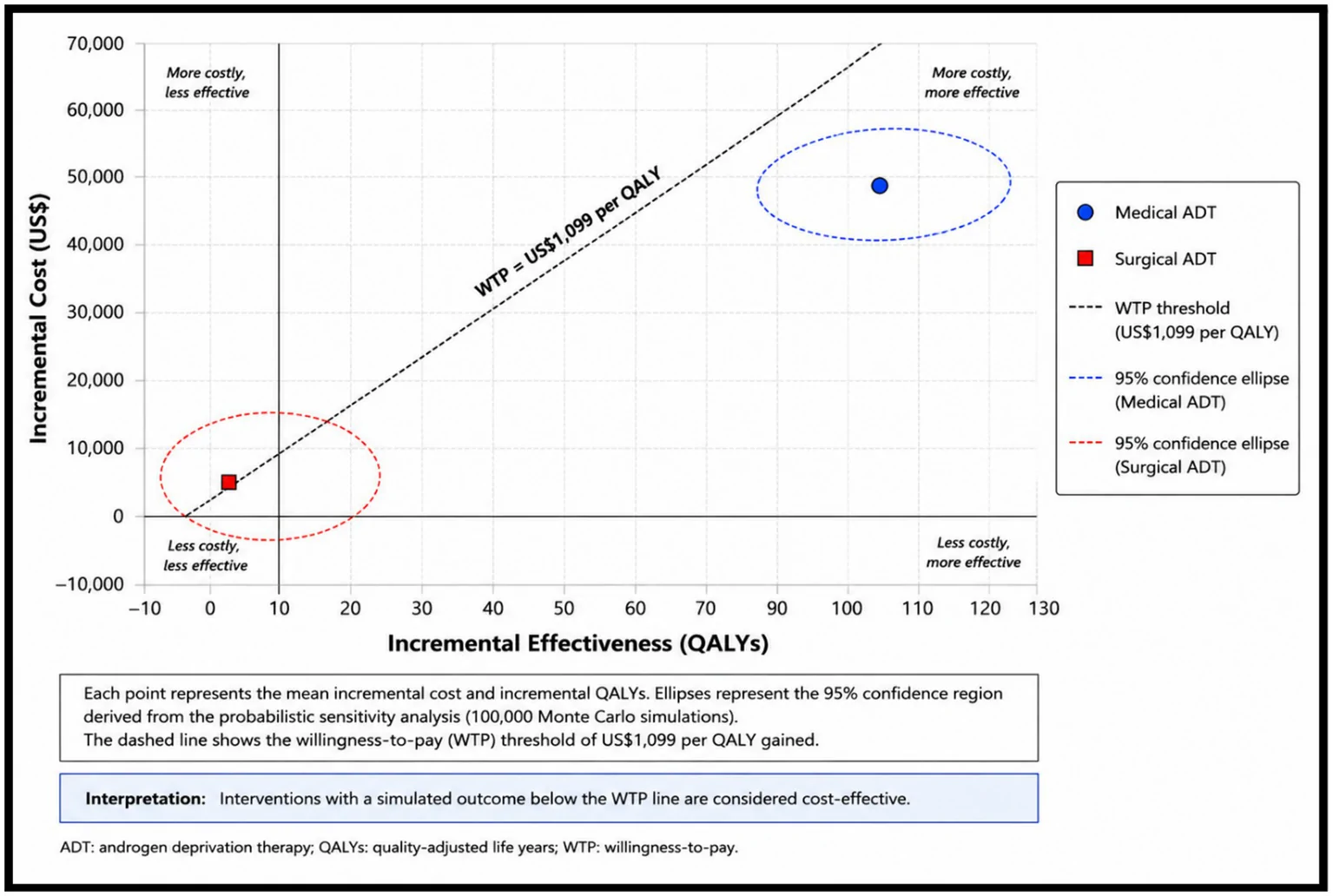
Cost-effectiveness plane (Medical ADT vs. Surgical ADT).

Figure 3 presents the cost-effectiveness acceptability curve derived from probabilistic sensitivity analysis. The probability that medical ADT would be considered cost-effective increased with higher WTP thresholds. At the GDP-based WTP scenario of US$1,099 per QALY gained, Figure 3 indicates a probability of >99% that medical ADT is cost-effective. The cost-effectiveness plane demonstrated that medical ADT was associated with higher expected costs but also greater health benefits than surgical ADT, placing the intervention in the north-east quadrant. This indicates that medical ADT is more effective but incurs additional costs. However, because the incremental cost-effectiveness ratio (US$430.44 per QALY gained) remained substantially below Tanzania’s willingness-to-pay threshold (US$1,099 per QALY gained), the additional health gains were considered economically worthwhile.

**Figure 3:**
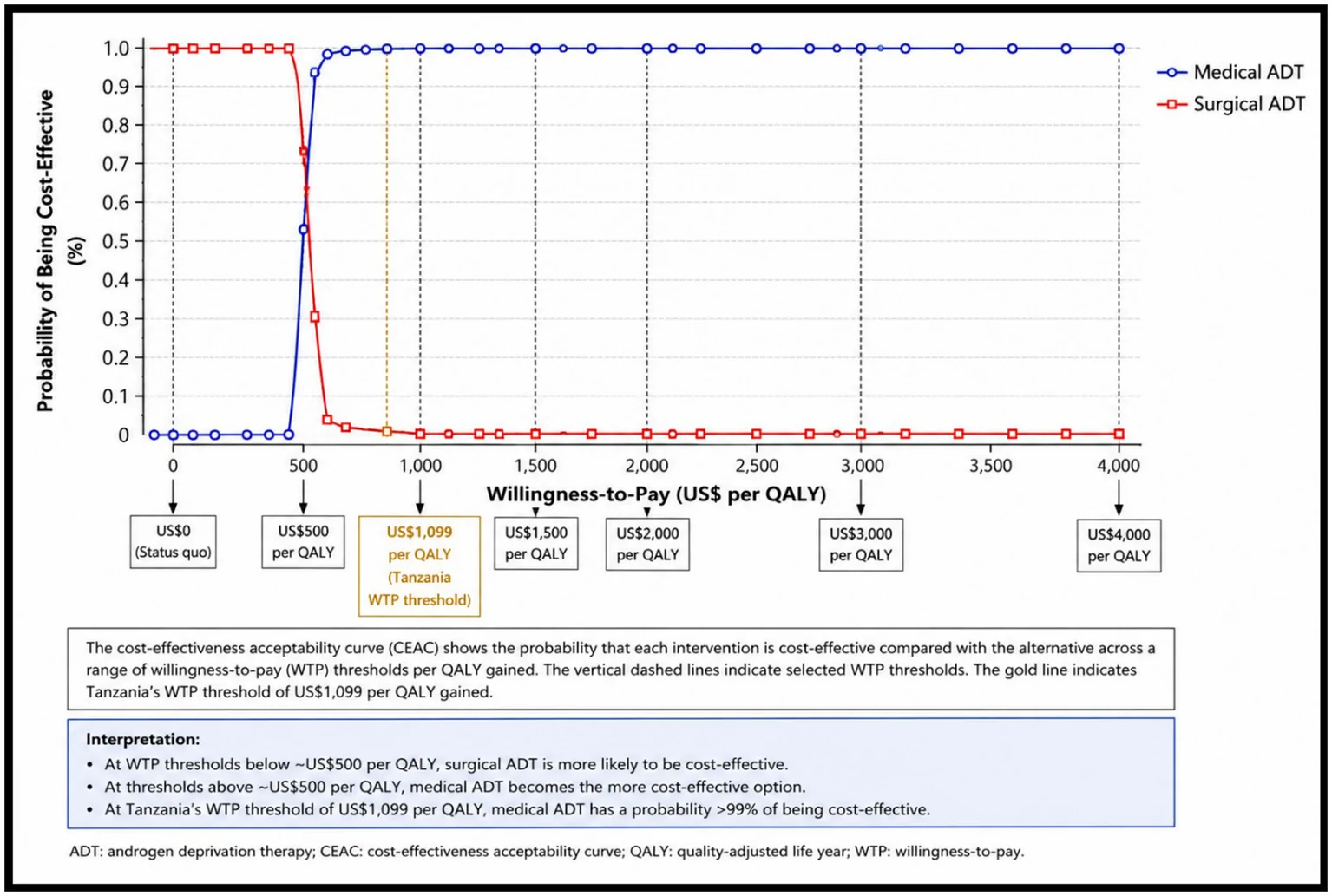
The distribution of CE values of Medical ADT or Surgical ADT interventions accepted at a set WTP value.

The probabilistic sensitivity analysis scatter plot is presented in Figure 4. Most simulation iterations were concentrated in the north-east quadrant of the cost-effectiveness plane, indicating that medical ADT consistently generated greater effectiveness at higher costs than surgical ADT. The limited overlap observed between the interventions suggests that the model results were relatively stable across simulated parameter values.

**Figure 4:**
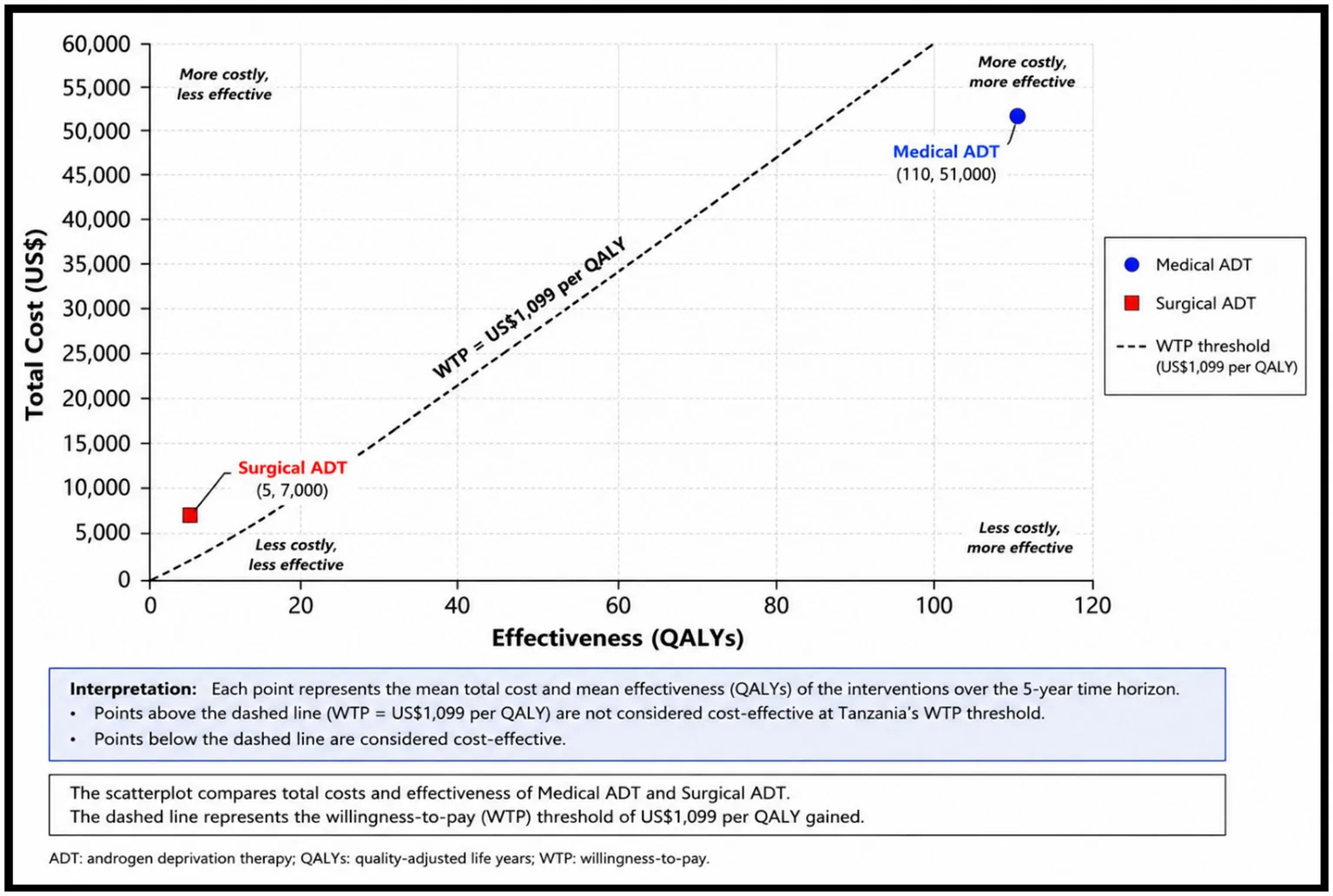
Probabilistic sensitivity analysis of incremental costs and incremental QALYs for medical ADT versus surgical ADT.

Each point represents one Monte Carlo simulation. The diagonal line represents the GDP-based WTP scenario of US$1,099 per QALY gained. Points below the WTP line indicate simulations in which the incremental cost per QALY gained was below the specified WTP scenario. The total QALYs reported represent the cumulative discounted health benefits accrued across all annual Markov cycles over the five-year analytic horizon. These values were estimated by combining the time spent in each health state with the corresponding utility values applied in the model.

### Sensitivity analysis results

Probabilistic sensitivity analysis was conducted using second-order Monte Carlo simulation with 100,000 interactions to assess the robustness of the base-case findings. As shown in Figure 2, most simulation iterations were located in the north-east quadrant of the cost-effectiveness plane, indicating that medical ADT was generally associated with higher costs and greater effectiveness than surgical ADT. The cost-effectiveness acceptability curve (Figure 3) demonstrated that the probability of medical ADT being cost-effective increased with higher WTP thresholds. At the base-case WTP threshold of US$1,099 per QALY gained, medical ADT remained the preferred strategy in the majority of simulations. Similarly, the scatter plot (Figure 4) showed that most simulated ICER estimates remained below the WTP threshold, suggesting that the conclusion regarding the cost-effectiveness of medical ADT was robust to uncertainty in model parameters.

## Discussion

This study evaluated the cost-effectiveness of medical ADT compared with surgical ADT among patients with advanced prostate cancer in Tanzania using a Markov decision-analytic model. The findings indicate that medical ADT generated greater health benefits than surgical ADT, although at substantially higher costs. Despite the additional expenditure, the estimated ICER remained below the selected WTP threshold, suggesting that medical ADT represents a cost-effective use of healthcare resources for managing advanced prostate cancer in Tanzania. These findings are particularly relevant in settings where most patients present with advanced disease and treatment options are largely limited to androgen deprivation strategies.

The findings are consistent with previous economic evaluations conducted in other settings. Bayoumi et al. reported that medical androgen suppression therapies could achieve favorable cost-effectiveness profiles when quality-of-life outcomes were incorporated into economic analyses, despite their higher acquisition costs compared with orchiectomy (4). Similarly, Akaza concluded that medical androgen suppression may provide acceptable value for money when treatment effectiveness and patient preferences are considered (22, 23). More recent studies by Wang et al. and Barbier et al. (12, 14) demonstrated that treatments associated with improved survival and QALYs may remain economically attractive even when associated with substantially higher direct medical costs. Although both medical ADT and surgical ADT are intended to achieve androgen suppression and disease control, differences in patient preferences, treatment acceptability, and health-related quality of life may influence the quality-adjusted outcomes associated with each strategy. Although both medical ADT and surgical ADT achieve androgen suppression through different mechanisms, the two strategies are not expected to yield identical quality-of-life outcomes. Differences in treatment acceptability, reversibility, side-effect burden, and patient preference may influence health-related quality of life independently of disease control, which may explain the magnitude of the observed QALY difference. Consequently, the observed differences in QALYs may reflect quality-of-life effects rather than substantial differences in disease progression. This interpretation is consistent with the principles of cost-utility analysis, where treatment value is assessed not only in terms of survival but also in terms of health-related quality of life experienced by patients.

Although these studies were conducted in higher-income settings, our findings suggest that similar economic relationships may exist in resource-constrained settings such as Tanzania, where maximizing health gains from limited healthcare resources remains a priority. The medical and surgical ADT cost included the unit cost of the intervention/procedure and consultation fee per visit. Medical ADT included Goserelin and Bicaltumide, while Surgical ADT5 included Bilateral Subcapsular Orchiectomy (BSO) or Bilateral Total Orchiectomy (BTO). Hospitalization costs were included where documented; however, variability in hospitalization patterns and incomplete documentation may have resulted in underestimation of disease-management costs. The cost for infrastructure and utilities of the hospital, including salaries, electricity, and water bills, was not included. This increases the differences with assumptions that were taken into consideration in other studies (8, 22).

Furthermore, the study assumed that patients with advanced prostate cancer at a disease state of local recurrent and Asymptomatic distant metastatic can be treated by either medical or surgical ADT. Patients with symptomatic metastatic disease were modeled within their assigned treatment strategy; patients who received both medical and surgical ADT in routine clinical practice were excluded from the comparative economic analysis. Goserelin and Bicaltumide were the commonly used medical ADTs. Further studies are warranted to evaluate the cost-effectiveness of newer medical ADT regimens with closer monitoring of clinical response.

The uptake of surgical ADT was substantially lower than that of medical ADT, which may have introduced treatment-group imbalance and potential selection bias. This calls for more psychosocial support studies to improve the quality of life of patients with advanced prostate cancer, to address patients’ concerns as part of best practices for the value of money and enhance patients’ patient-centered approach to quality improvement (24–26). Incomplete documentation in some patient records may have introduced selection bias and limited the completeness of the available clinical and cost data. Probabilistic sensitivity analysis (PSA) was conducted using second-order Monte Carlo simulation with 100,000 iterations. Where published studies did not report standard deviations or confidence intervals for model parameters, uncertainty was represented by assigning appropriate probability distributions and plausible parameter ranges based on published literature and accepted health economic modeling practice. In the sensitivity analysis, we used the range of costs and effectiveness obtained from the literature (4). During each simulation, parameter values were randomly sampled from the assigned distributions to evaluate the joint uncertainty surrounding costs and health outcomes.

The present model intentionally adopted a simplified five-state disease progression framework to facilitate comparison of the long-term costs and health outcomes associated with surgical and medical androgen deprivation therapy. Although prostate cancer progression is influenced by factors such as Gleason score, tumour burden, and treatment-related complications, these variables were not explicitly modeled because the objective was to estimate overall cost-effectiveness at the population level rather than patient-specific prognosis.

The preference for medical ADT observed in this study may reflect psychosocial concerns associated with orchiectomy, including perceived loss of masculinity, stigma, and fear of permanent body image changes. Although surgical ADT is less costly, patients and providers may favor medical therapy because it is reversible and perceived as less psychologically distressing. These findings have important implications for patient-centered prostate cancer care and resource allocation in Tanzania.

The scenario comparison demonstrated that published utility estimates for advanced prostate cancer are relatively consistent across recent economic evaluations, suggesting that the selection of alternative utility sources would be unlikely to materially alter the comparative interpretation of the findings. Likewise, the disease progression pathway adopted in the present model is consistent with the natural history framework described by Bayoumi et al. and subsequent economic evaluations. Nevertheless, future studies using Tanzanian-specific utility estimates and longitudinal clinical data should further evaluate the robustness of these findings.

One important consideration when interpreting our findings is that several transition probabilities and health-state utility values were obtained from international literature because Tanzania currently lacks published longitudinal studies describing disease progression among patients with advanced prostate cancer and locally derived preference-based utility estimates. We selected studies with disease characteristics, treatment pathways, and health states that closely resembled those represented in our Markov model, thereby maximizing the applicability of the evidence to the Tanzanian setting. Although differences in supportive care, symptom management, and patient perceptions of health-related quality of life may exist between Tanzania and high-income countries, the biological progression of advanced prostate cancer is expected to be broadly comparable across settings. Furthermore, uncertainty surrounding these parameters was explored through sensitivity analyses, which demonstrated that the principal conclusions regarding the cost-effectiveness of medical ADT remained robust.

Although Goserelin and Bicalutamide were the most frequently prescribed medical ADTs, the present study evaluated medical ADT as a treatment strategy rather than individual drug regimens. Regimen-specific analyses would require larger patient cohorts and more detailed treatment-specific effectiveness, adverse event, and cost data than were available for the current study.

Beyond cost-effectiveness, equity considerations are important when interpreting these findings. Although medical ADT was found to be cost-effective compared with surgical ADT, its higher upfront treatment costs may present financial barriers for uninsured patients, particularly in settings where out-of-pocket payments remain common. Consequently, cost-effectiveness alone may not guarantee equitable access to treatment. This is consistent with the principles of Universal Health Coverage (UHC), which emphasize that access to essential health services should depend on need rather than the ability to pay and that financial risk protection is fundamental to equitable healthcare delivery (27). Expanding health insurance coverage and strengthening financial risk protection mechanisms, including the ongoing universal health insurance reforms in Tanzania, could improve access to medical ADT while reducing financial hardship among patients with advanced prostate cancer. The recently enacted Universal Health Insurance Act seeks to expand insurance coverage and improve equitable access to healthcare services across Tanzania (28). The findings concur with previous studies demonstrating that expanding health insurance coverage and strengthening equitable health financing mechanisms are essential to ensure that cost-effective health interventions are also financially accessible to all eligible patients, particularly vulnerable and uninsured populations (29).

### Health financing and policy implications

The findings have important implications for health financing and resource allocation in Tanzania. Although medical ADT was found to be cost-effective, its adoption may impose substantial budgetary demands because of the large number of patients presenting with advanced disease. From a policy perspective, the findings support the integration of economic evidence into national cancer control strategies and clinical practice guidelines. Given the increasing incidence of prostate cancer and the predominance of advanced-stage presentation in Tanzania, policymakers should consider strengthening access to cost-effective treatment options while simultaneously promoting earlier diagnosis and referral. Decision-makers should consider not only cost-effectiveness but also affordability and budget impact when planning prostate cancer services. Expansion of health insurance coverage and strategic purchasing mechanisms may improve access to cost-effective treatments while maintaining financial sustainability. Furthermore, incorporating patient preferences into treatment planning may improve treatment adherence and overall quality of care. The findings support ongoing efforts toward Universal Health Coverage in Tanzania by demonstrating that reimbursement of cost-effective cancer therapies through health insurance schemes may improve both health outcomes and equity in access to care.

Implementation considerations should also be taken into account when scaling up medical ADT in Tanzania. Injectable LHRH agonists such as goserelin require reliable cold-chain storage and handling, which may not be consistently available at lower-level health facilities. Successful decentralization of medical ADT would therefore require investment in cold-chain infrastructure, training of health workers to safely administer injectable therapy, and strengthened monitoring systems to detect and manage treatment-related adverse events. Without these supporting systems, the favorable cost-effectiveness of medical ADT observed at tertiary hospitals may not be fully realized at lower levels of the health system.

### Strengths and limitations of the study

This study has several strengths. First, it represents one of the few cost-effectiveness analyses of prostate cancer treatment strategies conducted in Tanzania and among the limited number reported from sub-Saharan Africa. Second, the study combined local hospital data with published evidence to develop a context-specific economic model relevant to decision-makers. Third, the analysis incorporated both cost and health outcomes, thereby providing a comprehensive assessment of value for money. Finally, sensitivity analyses were conducted to explore uncertainty surrounding key model parameters and to assess the robustness of the findings. However, this study had some limitations. First, some effectiveness and utility parameters were derived from international literature due to limited local Tanzanian data. Second, retrospective hospital records contained incomplete documentation for some variables. Third, the analysis may not fully capture all indirect and intangible costs associated with prostate cancer management. Another limitation, the findings of this study should be interpreted in the context of the study setting. Cost estimates were derived from two national referral hospitals, where patients typically present with more advanced disease and receive more specialized services than those available at regional, district hospitals or non-tertiary settings. Consequently, treatment costs and resource utilization may differ from those observed in lower-level healthcare facilities. While the biological effectiveness of ADT is unlikely to differ substantially across healthcare settings, the economic findings may not be directly generalizable to primary or secondary healthcare facilities in Tanzania.

A limitation of the economic model is that the original TreeAge Pro model file and its cycle-specific outputs were no longer accessible at the time of manuscript revision because the software license had expired. Consequently, although the model structure, parameter values and calculation approach could be documented, the original cycle-specific state occupancy and QALY calculations could not be independently reproduced. The reported cohort-level QALY estimates should therefore be interpreted as model-generated estimates conditional on the original model assumptions rather than as directly observed patient-level outcomes. Another limitation of the study is that the proportion of records excluded specifically because of missing information could not be retrospectively quantified because the original screening log was unavailable. Consequently, the potential magnitude of selection bias arising from incomplete documentation cannot be precisely assessed. Another limitation of this study is that the probabilistic sensitivity analysis used the original TreeAge Pro model file and detailed distribution parameters could not be retrieved following expiry of the software licence. Although the original analysis used beta and gamma distributions and 100,000 Monte Carlo simulations, the exact distribution parameters could not be independently verified during manuscript revision. The PSA findings should therefore be interpreted as exploratory evidence of decision uncertainty rather than as fully reproducible simulation estimates.

Finally, treatment-related adverse events were not explicitly modeled. Although adverse events such as hot flashes, osteoporosis, cardiovascular disease, and metabolic complications are recognized consequences of long-term ADT, reliable Tanzanian data describing their incidence, management costs, and quality-of-life impact were unavailable. Future studies should incorporate these outcomes as additional health states or temporary events within the Markov model to provide a more comprehensive assessment of treatment value.

## Conclusion

Medical ADT was found to be a cost-effective treatment strategy compared with Surgical ADT among patients with advanced prostate cancer in Tanzania. Medical ADT was associated with greater quality-adjusted health outcomes than surgical ADT, although at a higher cost. The estimated ICER remained below the selected willingness-to-pay threshold, suggesting that medical ADT represents a cost-effective treatment strategy for patients with advanced prostate cancer in Tanzania. The estimated ICER of US$430.44 per QALY gained was substantially below the selected willingness-to-pay threshold, indicating that medical ADT represents a cost-effective use of healthcare resources. These findings support consideration of medical ADT as a preferred treatment option for eligible patients with advanced prostate cancer within the Tanzanian healthcare system. Policy decisions regarding ADT should therefore consider not only cost-effectiveness but also affordability, financial protection, and equitable access, particularly for uninsured and socioeconomically disadvantaged populations. Further research should incorporate cost-effectiveness and survival, taking into consideration the health-related quality of life of patients, to strengthen the evidence base for advanced prostate cancer resource allocation decisions in Tanzania.

## Data Availability

The original contributions presented in the study are included in the article/supplementary material, further inquiries can be directed to the corresponding author.
